# Oxo-Rhenium-Mediated Allylation of Furanoside Derivatives: A Computational Study on the Mechanism and the Stereoselectivity

**DOI:** 10.1021/acs.joc.2c00393

**Published:** 2022-07-12

**Authors:** Emanuele Casali, Alessio Porta, Lucio Toma, Giuseppe Zanoni

**Affiliations:** Department of Chemistry, University of Pavia, Viale Taramelli, 12, 27100 Pavia, Italy

## Abstract

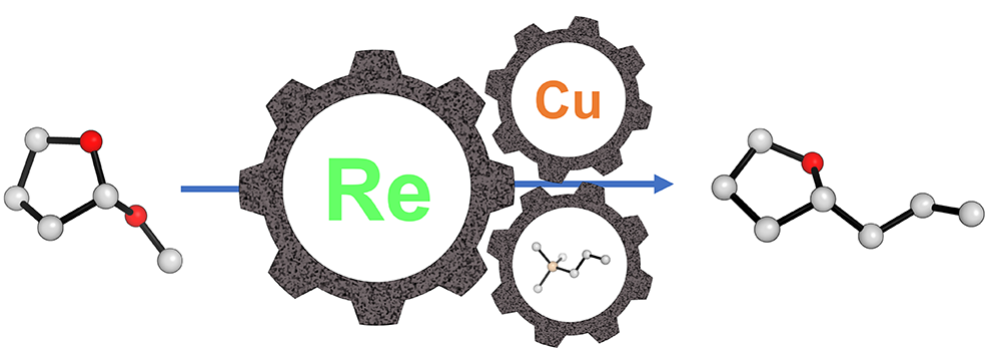

Properly substituted tetrahydrofuran (THF) rings are important building blocks in the synthesis of many natural metabolites. Having reliable procedures to control the stereoselectivity at the THF core while decorating it with different substituents is a fundamental requirement to achieve and fulfill the complexity of nature. We recently reported a new chemical approach to control the stereochemistry in the alkylation and arylation of furanoside derivatives by using a rhenium(V) complex to form an intermediate oxo-carbenium species able to react with proper soft nucleophiles. Here, we describe theoretical calculations, performed at the DFT B3LYP level, to disclose the important mechanistic features which regulate the entire catalytic cycle of the reaction of mono- and disubstituted furanosides with allyltrimethylsilane catalyzed by Re(O)Cl_3_(OPPh_3_)(Me_2_S). Moreover, the key factors governing the allylation step were investigated, confirming that the stereoselectivity, which is independent of the anomeric configuration of starting acetal, mainly arises from the orientation of the substituent at C-4, with only marginal contribution of the substituent at C-5. Finally, puckering Cremer–Pople parameters were used to take trace of the structural modifications throughout the catalytic cycle.

## Introduction

In the realm of natural metabolites, a place of primary importance is held by THF motifs.^1^ A combination of the way in which they can be decorated and the control of the stereochemistry on the five-membered ring has served as a formidable challenge for synthetical chemists. To control the chirality on the ring, many different strategies have been elaborated during the years, but one in particular resulted to be the most widely adopted: namely, the venerable Sakurai–Hosomi reaction, which operates on the acetal forms of the tetrahydrofurans by involving strong Lewis acids (e.g., TMSOTf, SnCl_4_, BF_3_·Et_2_O, or TiCl_4_) to prepare the cyclic five-membered oxo-carbenium ion, and soft, nucleophilic allylsilanes to afford the corresponding allylated THF product.^1,2^ However, the high moisture sensitivity of strong Lewis acids has limited the applicability of this method. This problem was addressed during the years and was overcome by mainly two different strategies reported by Yadav and Friestad.^3,4^ The first one suggested the usage of molecular iodine in CH_2_Cl_2_ at cryogenic temperatures, while the second one uses TiCl_4_ as a Lewis acid again but under milder reaction conditions. Recently, we reported a new chemical opportunity to control the stereochemistry by using a rhenium(V) complex as a Lewis acid additive to obtain a metal-oxo-carbenium complex.^5^ To the best of our knowledge, besides the specific analysis for the anisole derivative we reported in our previous paper,^5^ no computational studies have been reported regarding the complete catalytic cycle for this reaction.

Seminal work in 2014 from Woerpel and co-workers computationally demonstrated the allylation step on five-membered ring acetals bearing fused rings, showing how subtle structural changes of the fused ring can result in dramatic influences on selectivity.^6^ Moreover, in 2021, the computational evidence of glycosyl cations as intermediates in the glycosylation reactions taking place through a S_N_1-type mechanism has been shown, thus confirming the validity of using this structural representation to describe the reaction.^7^

In the present work, we computationally investigate each step of the catalytic cycle promoted by the rhenium(V) catalyst in the allylation of furanoside derivatives, disclosing the key features which finely regulate the reaction mechanism and, importantly, the selectivity in the allylation step. Moreover, we dedicate special attention to the puckering parameters associated with the conformational changes the structures undergo during the course of the reaction.

## Computational Methods

The mechanism for the rhenium-catalyzed reaction was investigated using the Gaussian 09 program package^8^ in the framework of the density functional theory (DFT). All the calculations were carried out using, as a hybrid functional for DFT calculation, the Becke three-parameter hybrid exchange functional (B3) in its variation provided by the Lee, Yang, and Parr correlation functional (LYP).^9^ The basis set was specifically selected for each atom, considering the type and neat differences in the electronic distribution. Specifically, H, C, O, and F atoms were described by using the 6-311+G(d,p) basis set, whereas P, S, and Cl atoms were described using the 6-311+G(2df,p) basis set.^10,11,18^ Differently, the LanL2DZ effective core potential basis set was selected for the Re and Cu atoms.^12^ Open-shell calculations using the UB3LYP functional were performed when Cu(II) species were involved, to fulfill their doublet spin-state nature. Further calculations on benchmarking of theory can be found in the Supporting Information. For each structure, frequency calculations were performed to confirm the effective minimum or transition-state (TS) nature of the optimized structure. Moreover, intrinsic reaction coordinate (IRC) calculations were also performed to confirm the continuity of the reaction profile from TSs toward reactants, intermediates, or products.^13^ To reproduce the effect of the dichloromethane as a solvent, single-point calculations were performed by using the polarizable continuum solvent model (PCM).^14^ The final reported energies were then corrected to the zero-point energy: all the next evaluations will be referred to this level of theory. Analysis of the puckering parameters accompanying the conformational changes at the five-membered ring was also performed to identify the key conformational features regulating the stability of the intermediates to the final products.^15^

### Catalytic Cycle

**Scheme 1:**
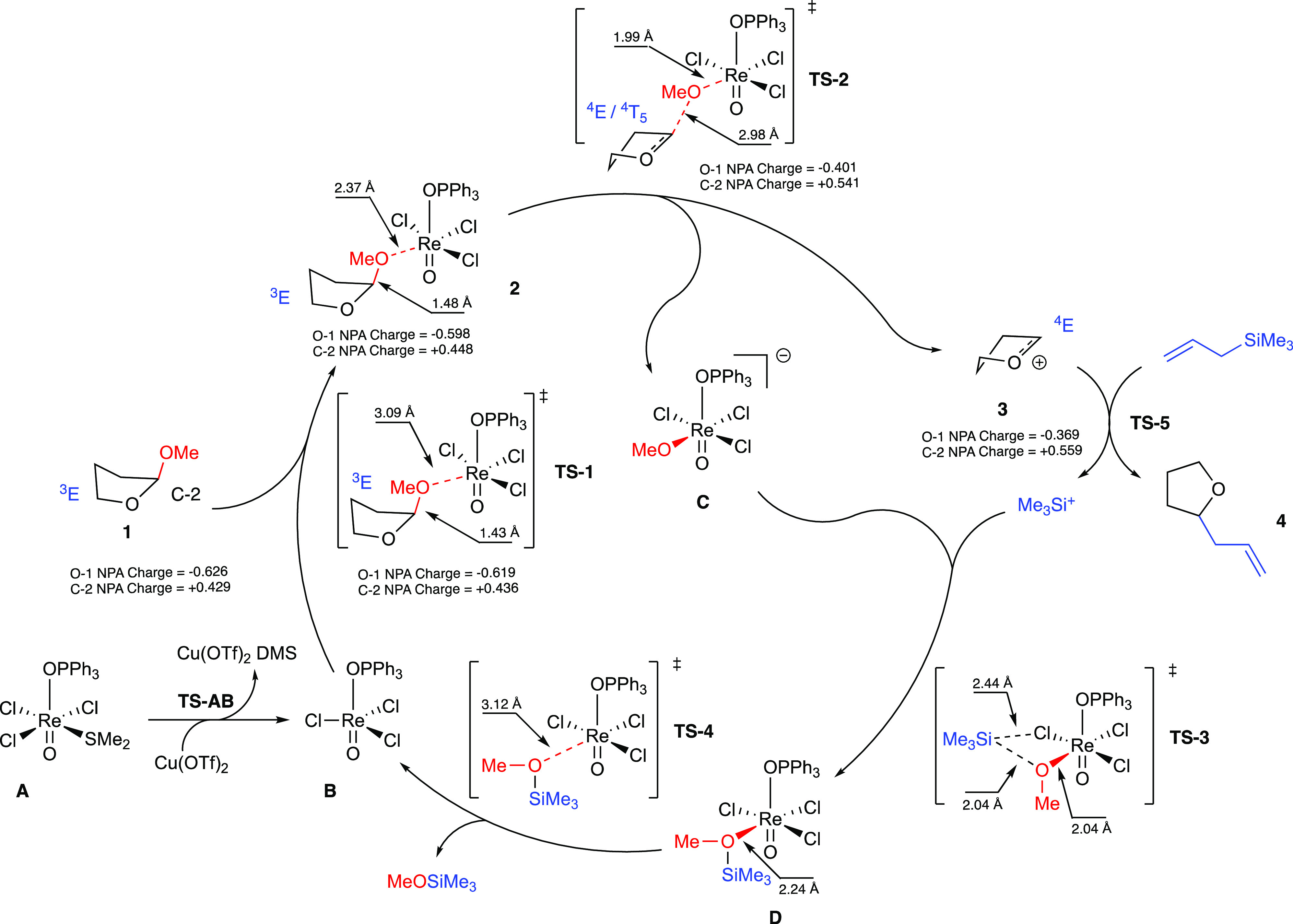
Proposed Mechanism for the Oxo-Re(V)-Catalyzed Allylation Based on DFT Calculations

To gain a better understanding of the mechanism of the oxo-Re(V)-mediated allylation reaction, DFT calculations were carried out by using a model system. Specifically, we used the 2-methoxytetrahydrofuran **1** as a precursor for the oxo-carbenium ion, while no simplifications were performed in the case of the rhenium catalyst **A**. Moreover, natural population analysis was performed, considering the charge stabilization for the oxygen (O-1) and the acetal carbon (C-2) atoms during the formation of the oxo-carbenium cation and the allylation process.^16^ The key steps of the mechanism are reported in Scheme 1, together with the important features we highlighted during the calculations. The analysis of the mechanism will be divided into four main sections that retrace the key steps of the reaction: the coordination of the THF acetal by the rhenium active species, the formation of the oxo-carbenium ion, its allylation, and the regeneration of the catalyst.

We also dedicated a special effort to model the formation of the catalytically active Re(V) species **B** from the rhenium catalyst **A**.

**Chart 1:**
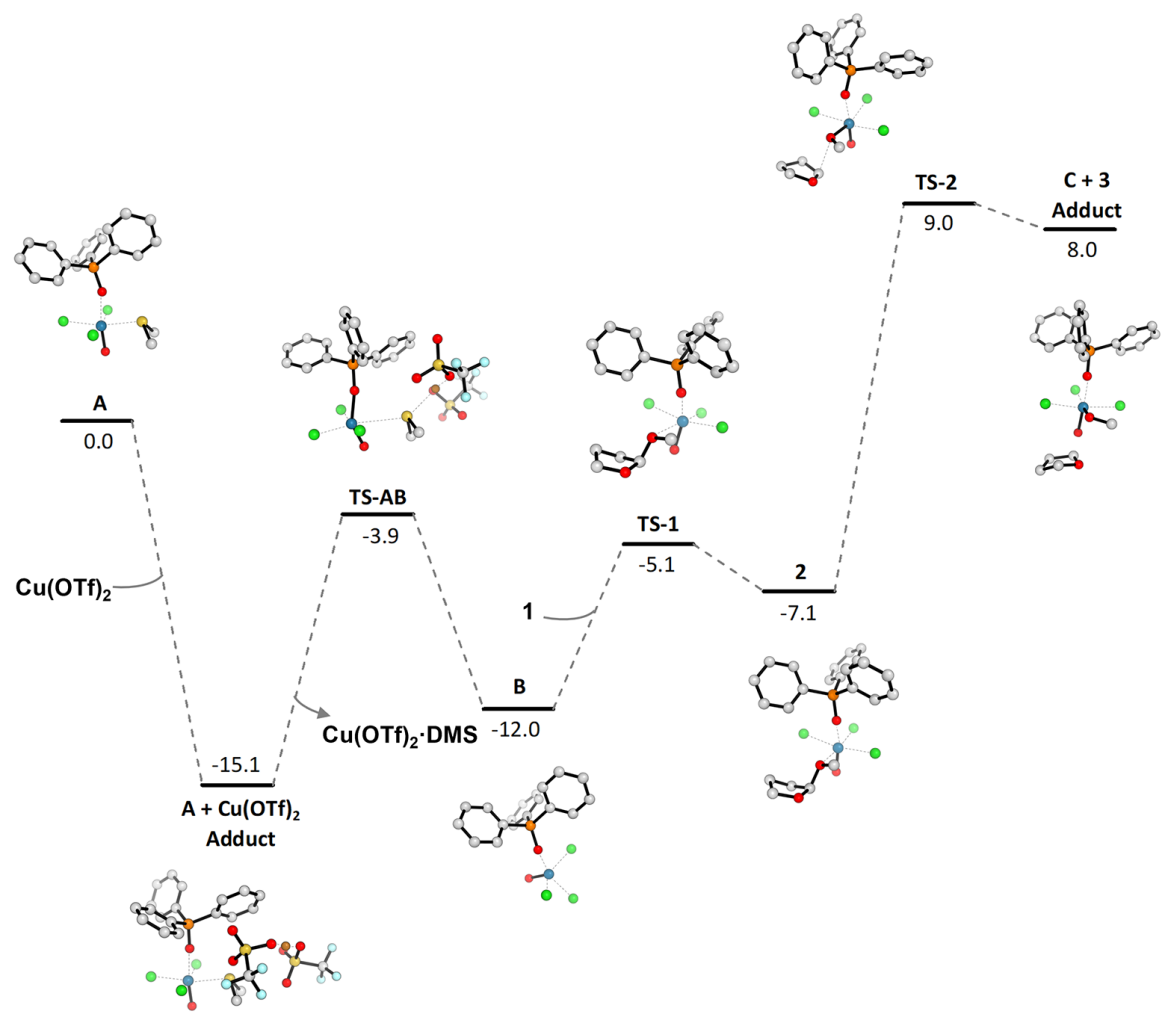
Electronic Energy Profile with ZPE Correction (kcal/mol) for the Reaction of DMS Displacement from Re Catalyst **A** to **B** and the Next Coordination of 2-Methoxytetrahydrofuran **1** up to the Formation of Oxo-Carbenium **3**a

Indeed, the reaction initiates with the Cu(OTf)_2_ displacement of Me_2_S from the rhenium complex Re(O)Cl_3_(OPPh_3_)(Me_2_S) **A**. This reaction step was investigated computationally, showing an activation energy for the ligand exchange of 11.2 kcal mol^–1^ from the adduct between the two reagents **A + Cu(OTf)**_**2**_**Adduct**, with an increase of stability for the obtained **B** product of nearly 12 kcal mol^–1^ from the starting materials (Chart 1). The driving force for this reaction step is due to the higher thiophilicity of Cu compared with that of Re.

a (U)B3LYP/6-311+G(d,p)&6-311+G(2df,p)&LanL2DZ/CH_2_Cl_2_(PCM)//(U)B3LYP/6-311+G(d,p)&6-311+G(2df,p)&LanL2DZ.

#### Coordination of THF-Methoxy Acetal **1** by the Reactive Re Species **B**

The catalytically active *12e*^*–*^ Re-complex **B** thus formed reacted with the 2-methoxytetrahydrofuran **1** to produce a THF–Re complex **2** via a low-barrier (6.9 kcal mol^–1^) TS, **TS-1** (Chart 1). During this step, an almost unchanged charge on the C-2 carbon of the THF ring (from +0.429 to +0.436) has been observed; the same situation occurs also on the O-1 oxygen, which showed an unaltered value of charge (from −0.626 to −0.619) but of the opposite sign (Scheme 1). In **TS-1**, substrate **1** directs only the aglycone oxygen atom of the −OMe residue toward the Re metal center by maintaining the more stable *exo*-anomeric orientation for the methyl group during the formation of the interaction (3.09 Å). The THF–oxygen remains far from the Re coordination sphere; in fact, when we considered a possible chelation of the two oxygen atoms of **1** to rhenium, less stable structures were found both for the complex **2** and **TS-1**. This particular behavior was observed for all further steps of the catalytic cycle and thus has been excluded as a viable pathway.

The Re–OMe distance registered during the TS (3.09 Å) was significantly shortened to 2.37 Å in the intermediate **2**. Moreover, the evaluation of puckering parameters showed no considerable modification in conformation by moving from **1** to **TS-1** and finally to **2**: the ^3^*E* envelope conformation remains unchanged during all these steps (see the Puckering polar plot in the Supporting Information for more details), as well as the C-2/OMe distance only slightly lengthened from 1.41 Å in **1** to 1.43 Å in **TS-1** and 1.48 in **2**. This indicates that no modification in the structure of the THF acetal is necessary during the direct coordination of the -OMe substituent to the rhenium metal center (see Table S2 in the Supporting Information for more details). Another important detail that arises from these calculations is related to the pyramidalization of the C-2 atom, which indirectly indicates the hybridization at C-2. To describe this property, we decided to look for the sum of the angles between the substituents at C-2 not involved in the reaction (namely, O-1, H, and C-3). By measuring the angle between them and summing the results, we can conclude that if the sum is close to 360°, the hybridization at C-2 is almost sp^2^, while if the sum is close to 330°, the hybridization at C-2 is almost sp^3^. The results we got go from 327.0° in **1**, to 328.1° in **TS-1**, and then to 329.5° in **2**, thus indicating that **TS-1** has the only role in coordinating **1** to the Re-complex with no change in the hybridization at C-2, with the angle values being compatible with sp^3^ hybridization of C-2 (see Table 1).

**Table 1:**
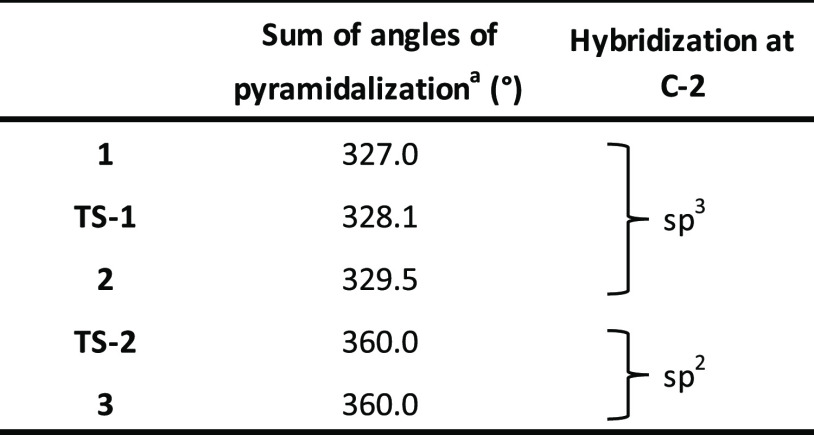
Pyramidalization Angles Related to the Hybridization at the C-2 Atom for the Catalytic Cycle

a Sum of all angles on C-2 (O1-C2-H + O1-C2-C3 + C3-C2-H).

#### Formation of Oxo-Carbenium Ion **3**

Complex **2** then moves toward the rate-determining **TS-2** with an energy barrier of 16.1 kcal mol^–1^. **TS-2** features an increased positive charge on C-2, from +0.448 in **2** to +0.541, and a significantly less negative charge on O-1, from −0.598 to −0.401. The complete breaking of the crucial MeO–THF bond, increasing in distance from 1.48 to 2.98 Å, and the concomitant shortening of the MeO–Re distance to 1.99 Å indicate a product-like nature of **TS-2**. Once the energy barrier has been overcome, two charged species are formed, namely, the cationic oxo-carbenium **3** (charges of +0.559 and −0.369 for C-2 and O-1, respectively) and the anionic rhenium complex **C**. This step also highlights the S_N_1 mechanism of this reaction: the slow ionization to form the oxo-carbenium ion is achieved in **TS-2** during the rate-determining step, and only in a second time, the fast reaction between the intermediate cation and the nucleophile occurs (see Chart 3).

During this step, the THF ring reverts the envelope conformation from ^3^*E* in **2** to ^4^*E* in **3**, passing through an intermediate conformation during **TS-2**, which can be described as ^4^*E*/^4^*T*_5_ (see Table S2 and the Puckering polar plot in the Supporting Information for more details). This observation is in accordance also with the change in the pyramidalization of the C-2 atom which depends on the hybridization of C-2: the sum of angles moves from 329.5° in **2** to 360.0° in **TS-2**, a compatible value with the sp^2^ hybridization of C-2 in **3** (see Table 1).

Further interaction of **3** with allyltrimethylsilane afforded the corresponding allylated THF **4** and trimethylsilyl cation, a reaction step that will be analyzed in the next sections by paying particular attention to the importance of the substituents on the THF core to explain the observed experimental selectivities.

#### Regeneration of Re Reactive Species **B**

**Chart 2:**
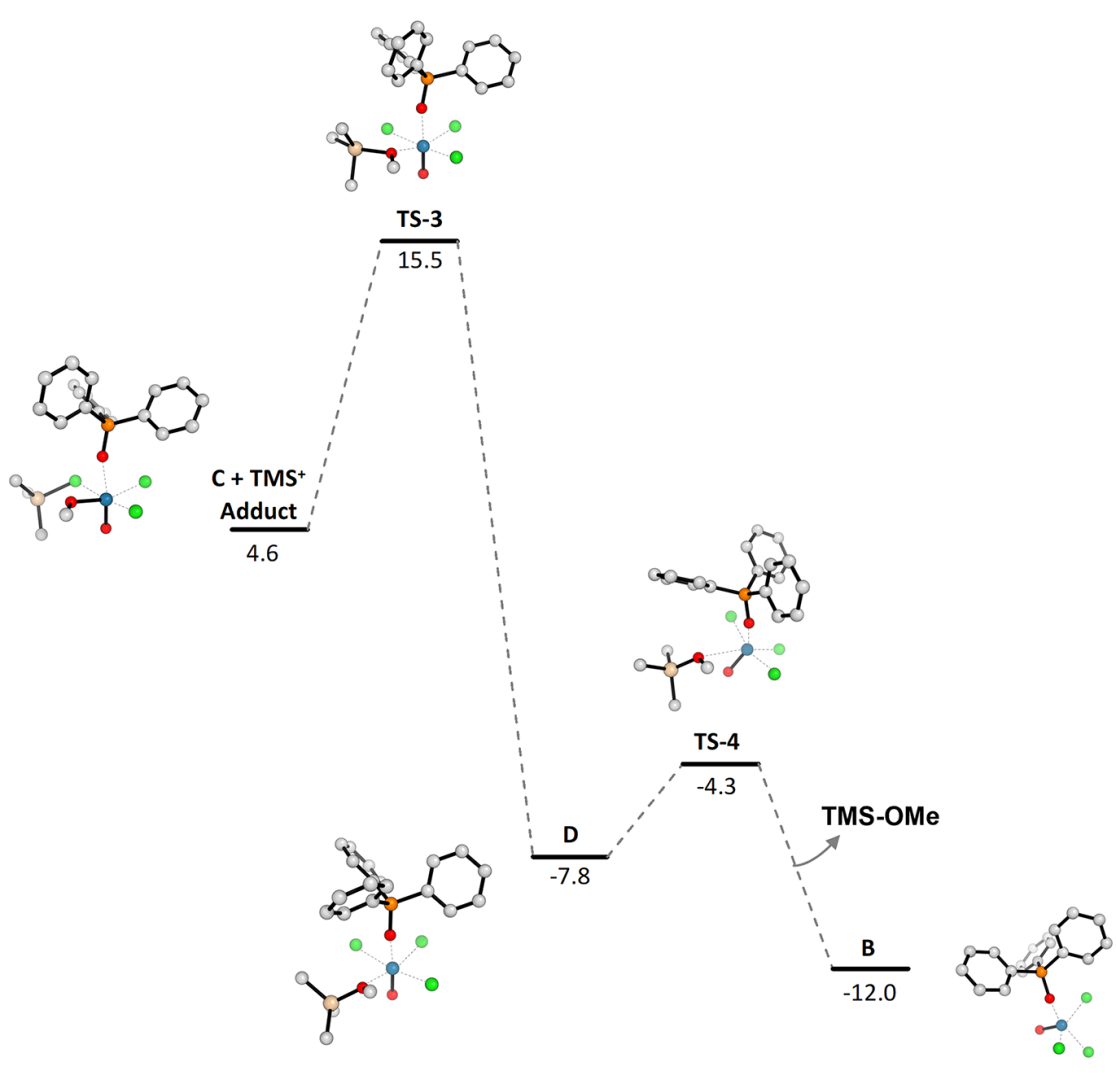
Electronic Energy Profile with ZPE Correction (kcal/mol) for the Regeneration of Re Catalyst Species **B**a

The trimethylsilyl cation, which originated from the allylation step, combines with anionic complex **C** during the neutral TS, **TS-3** (Chart 2). The incoming Si-cation directs toward the oxygen atom of the coordinated MeO-residue, with the assistance of the nearby coordinated chlorine atom. This transformation shows an energy barrier of 10.9 kcal mol^–1^ (**TS-3**) with the silicon atom shared between chlorine and oxygen atoms (bond lengths of 2.44 and 2.04 Å, respectively). The **TS-3** evolves in the next Re-complex **D**, with a completely formed Si–OMe bond (1.74 Å) and a stabilization of nearly 23 kcal mol^–1^ from the previous TS (Chart 2). Decomplexation of the MeO–TMS ether from the Re-complex **D** restores the *12e*^*–*^ Re-complex **B**, which re-enters the catalytic cycle. This last step occurs through the **TS-4** TS, with an activation energy of only 3.5 kcal mol^–1^.

a B3LYP/6-311+G(d,p)&6-311+G(2df,p)&LanL2DZ/CH_2_Cl_2_(PCM)//B3LYP/6-311+G(d,p)&6-311+G(2df,p)&LanL2DZ.

#### Allylation of Oxo-Carbenium Ions **3** and **5–8** with Allyltrimethylsilane

Locating the TS for the nucleophile addition to the oxo-carbenium species has been described in the literature to be a challenging study, which in the case of oxygen nucleophiles ended in a stalemate.^17^ However, by adopting our strategy and the below-reported computational procedure, we were able to locate the allylation step, which is fundamental to explain the observed experimental selectivity.

The computational investigation of the allylation of oxo-carbenium species **3** was performed by using a similar approach with respect to the other steps of the catalytic cycle. The corresponding TSs were still located through DFT calculations within B3LYP theory, by using the following differentiated basis set, that is 6-311+G(d,p) for the H, C, and O atoms^10,11a,18a^ and 6-311+G(2df,p) for the Si atom.^10,11b−11d,18b^ Differently from the previous approach, the solvent effect was already considered during the optimizations by using the PCM for dichloromethane, thanks to a smaller and simpler system to be investigated.^14^ The reported energies are corrected by the zero-point energy. In the case of substituted oxo-carbenium ions, the theoretical product distributions were obtained by applying Boltzmann population analysis at the corresponding experimental reaction temperature (see below).

**Chart 3:**
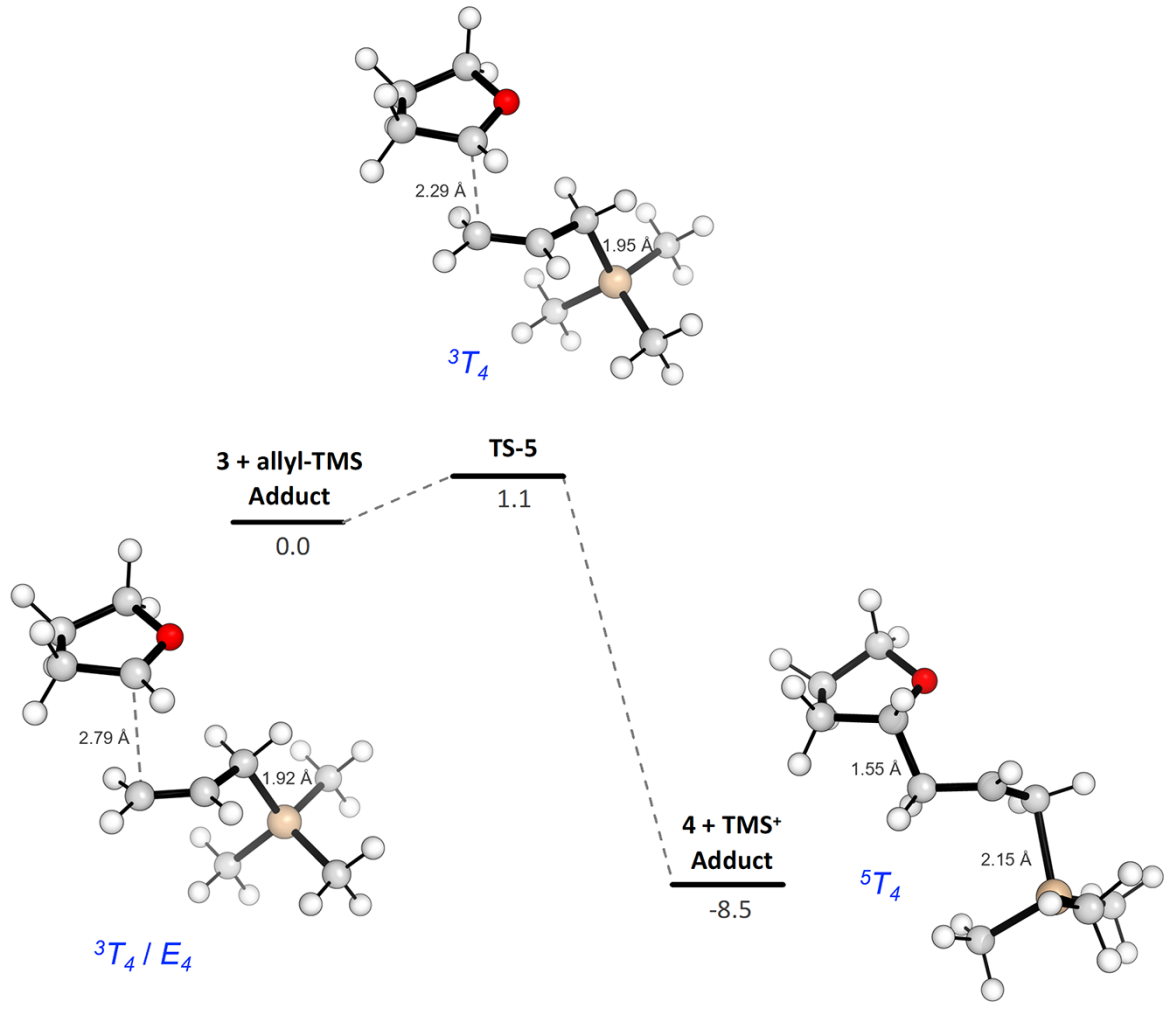
Electronic Energy Profile with ZPE Correction (kcal/mol) for the Allylation of Oxo-Carbenium ion **3**a

The allylation process on the simple ion **3**, which does not bear any substituents on the THF ring, shows an activation energy barrier of only 1.1 kcal mol^–1^ from the adduct of **3** with allyltrimethylsilane to the corresponding **TS-5**. The distances observed in this TS for the forming C–C and the breaking C–Si bonds (Chart 3) indicate a very asynchronous process. The formation of the new C–C bond (from 2.79 to 2.29 Å in **TS-5** and to 1.56 Å in the final adduct of **4** with trimethylsilyl cation) precedes the breaking of the C–Si bond (from 1.92 to 1.95 Å in **TS-5** and to 2.15 Å in the final adduct of **4** with trimethylsilyl cation). The corresponding change in the puckering parameters of the five-membered ring shows a conformation intermediate between *twisted* (^*3*^*T*_*4*_) and *envelope* (*E*_*4*_), which evolves to the *twisted* (^*3*^*T*_*4*_) in **TS-5** and finally to the *twisted* (^*5*^*T*_*4*_) conformation (see Table S3 in the Supporting Information for more details).

a In blue are reported the conformational descriptors for the five-membered ring. CH_2_Cl_2_(PCM)-B3LYP/6-311+G(d,p)&6-311+G(2df,p).

The pyramidalization of the C-2 atom was considered also in this step of the reaction by following the same procedure reported before. Specifically, we were able to observe the sum of angles moving from 359.0° in **3 + allyl-TMS Adduct**, to 352.8° in **TS-5**, and finally to 326.9° in **4 + TMS**^**+**^**Adduct**, perfectly corresponding to the transition from a sp^2^ hybridization of C-2 again to sp^3^ (see Table 2).

**Table 2:**
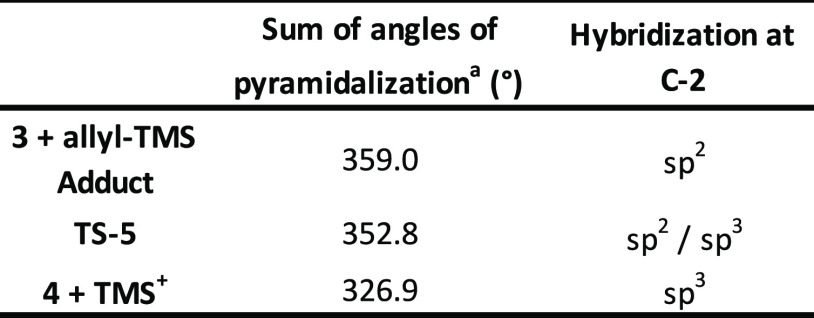
Pyramidalization Angles Related to the Hybridization at the C-2 Atom for the Allylation Step

a Sum of all angles on C-2 (O1–C2–H + O1–C2–C3 + C2–C3–H).

**Figure 1:**
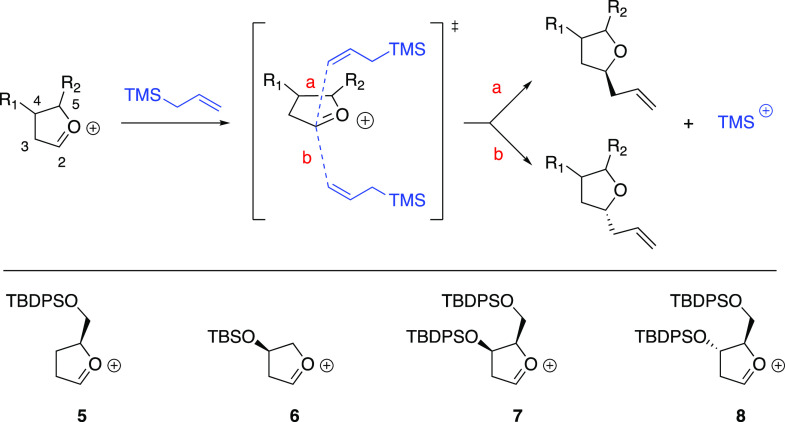
Allylation of oxo-carbenium species **5–8**.

We then moved to the substituted oxo-carbenium ions **5–8** which are the cationic intermediates of reactions described in either our previous paper^5^ or taken from the literature.^19a,20^ Specifically, we considered different situations depending on the substituents at C-4 and C-5 of the oxo-carbenium ion: we started with the mono-substituted glycosyl cations **5** and **6** to then increase the complexity of the system to disubstituted intermediates **7** and **8** (Figure 1). However, to accelerate the calculation without losing steric information, we decided to simplify the TBS or TBDPS moieties with a TMS group. Moreover, for species **5**, **7**, and **8**, since there is the possibility of different orientations of the substituent at the C-5 position, we evaluated all the three staggered rotamers by reporting in the tables each of the minima as **T1** (*gg-rotamer*), **T2** (*gt-rotamer*), and **T3** (*tg-rotamer*) (see Figure S1 in the Supporting Information for more details).^21^ Finally, the best conformer for each of the two possible attacks on the oxo-carbenium ion will be represented in a tridimensional structure in order to highlight the important features which regulate the observed selectivity.

According to the published experimental results,^3,19^ the stereochemistry of the anomeric center of the furanoside which originates the oxo-carbenium species is irrelevant to the observed selectivity; thus, only the allylation step determines the selectivity. In fact, it has been demonstrated by Woerpel and co-workers that the allylation step along the reaction pathway is governed by the Curtin–Hammett principle.^20^ Hence, for these substituted substrates, only the corresponding stereoisomeric TSs were located and the theoretical diastereomeric product ratio was determined by the relative energies of the two TSs (i.e., **a** and **b**) originating from the common oxo-carbenium intermediate (Figure 1).

**Figure 2:**
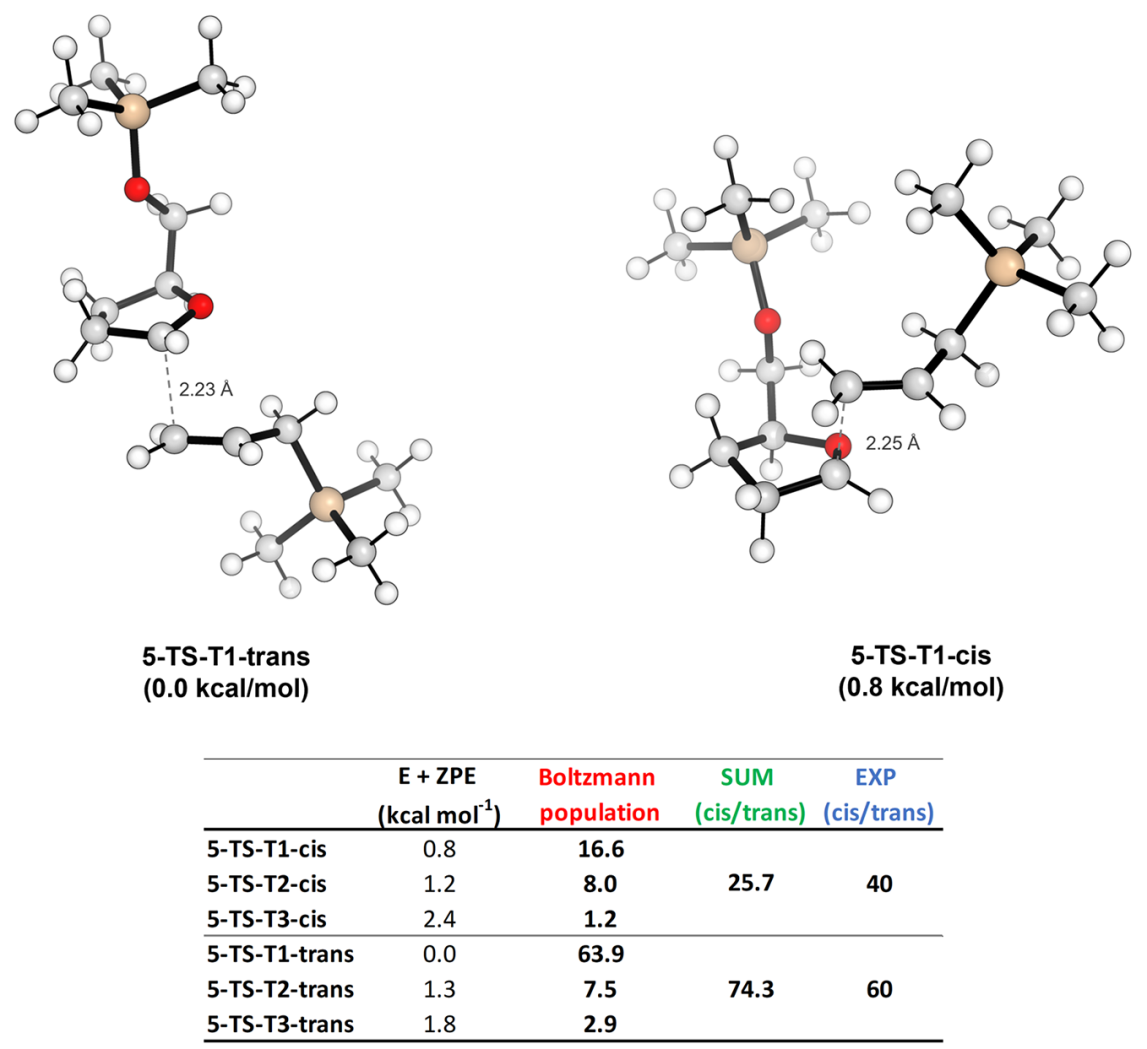
TSs for the trans- and cis-approach of the allyltrimethylsilane on **5** and the table reporting the Boltzmann percentage population evaluated at 22 °C for all possible conformations.

For the C-5 mono-substituted oxo-carbenium ion **5**, DFT calculations identified three TSs leading to the *trans*-2,5-disubstituted product and three TSs leading to the cis-product. The most stable TS of each approach (Figure 2) shows the oxygen of the side chain above the ring and close to the oxonium center to allow a stabilizing interaction with the oxo-carbenium moiety (**T1** conformation).

The selectivity appears to be strongly dependent on steric hindrance provided by the silylether group at C-5. In order to minimize the steric interaction with the pentacyclic ring, the TMS group of the ether substituent at C-5 is oriented outward. Thus, both diastereotopic faces of ion **5** are accessible, but the shielding of the upper face due to the inward OTMS group orientation results in a calculated energy difference between the two most populated TSs of 0.8 kcal mol^–1^, corresponding at 22 °C to a 74.3:25.7 trans-/cis-product ratio. This selectivity is in agreement with the experimental 60:40 value observed in the allylation of compound **5**, as reported by Woerpel and co-workers.^19a^ The distance between the oxo-carbenium C-2 carbon and the terminal allylic one resulted to be nearly the same in both the approaches (∼2.2 Å). Moreover, the allyltrimethylsilane distorts the structure of the oxo-carbenium ion in a different way, according to the direction of its approach: the trans-approach favors the ^*3*^*E*/^*3*^*T*_*4*_ puckering, while the *cis*-one prefers the opposite ^*4*^*T*_*3*_ (see Table S4 in the Supporting Information for more details).

**Figure 3:**
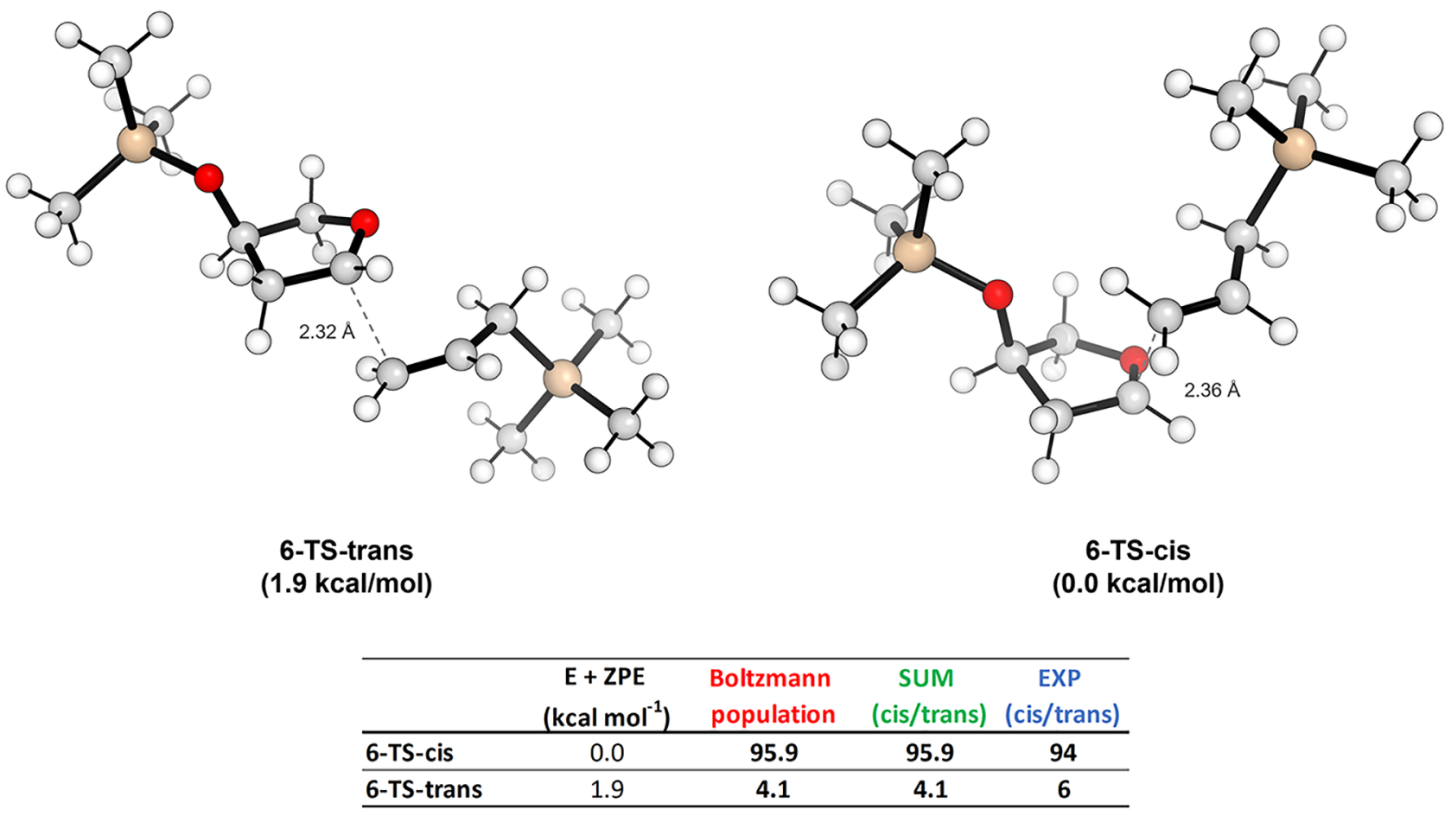
TSs for the trans- and cis-approach of the allyltrimethylsilane on **6** and the table reporting the Boltzmann percentage population evaluated at 23 °C.

Regarding the C-4 mono-substituted oxo-carbenium ion **6**, we do not have the three possible orientations reported for the case above since there is no methylene functionality that can result in an easier rotation of the dihedral angle. For this reason, we analyzed only one conformer for both the cis- and trans-approach. In both the situations, we observed that the silylether group at C-4 is oriented outward to reduce the steric hindrance. Moreover, in agreement with Woerpel’s observations,^19^ in both the TSs, the C-4 silylether group is pseudoaxially oriented (Figure 3). When the conformations with a pseudoequatorial substituent at C-4 were investigated, they were found not to be the local minima on the potential energy surface by converging to the pseudoaxial structures during the optimizations. The calculated energy difference between the two TSs is 1.9 kcal/mol, corresponding at 23 °C to a 95.9:4.1 cis/trans product ratio. This result is in good agreement with the experimental value of 94:6 determined for the allylation of **6**, as reported by Woerpel and co-workers.^19b,20^ In this new case, the distance between the oxo-carbenium C-2 carbon and the terminal allylic one resulted to be slightly longer than the previous one, yet is nearly identical in both approaches (∼2.3 Å). Also, in this case, the approach of the allyltrimethylsilane distorts the structure of the oxo-carbenium but not in an opposite manner for the two directions of the approach like in the previous situation. The trans-approach favors the *E*_*5*_/^*4*^*T*_*5*_ puckering, while the cis-one prefers the ^*4*^*T*_*3*_ like in the previous system (see Table S5 in the Supporting Information for more details). This variation is mainly due to the different location of the substituent on the oxo-carbenium structure, together with the higher conformational rigidity imparted by the pseudoaxially oriented silylether group at C-4.

The computational and experimental results on the allylation of the above reported oxo-carbenium ion intermediates clearly indicate the ability of the substituent at C-5 to promote the trans-allylation, whereas the alkoxy substituent at C-4 is significantly cis-orienting. These abilities may result in a match or mismatch when the 4,5-disubstituted oxo-carbenium ions **7** and **8** are considered.

**Figure 4:**
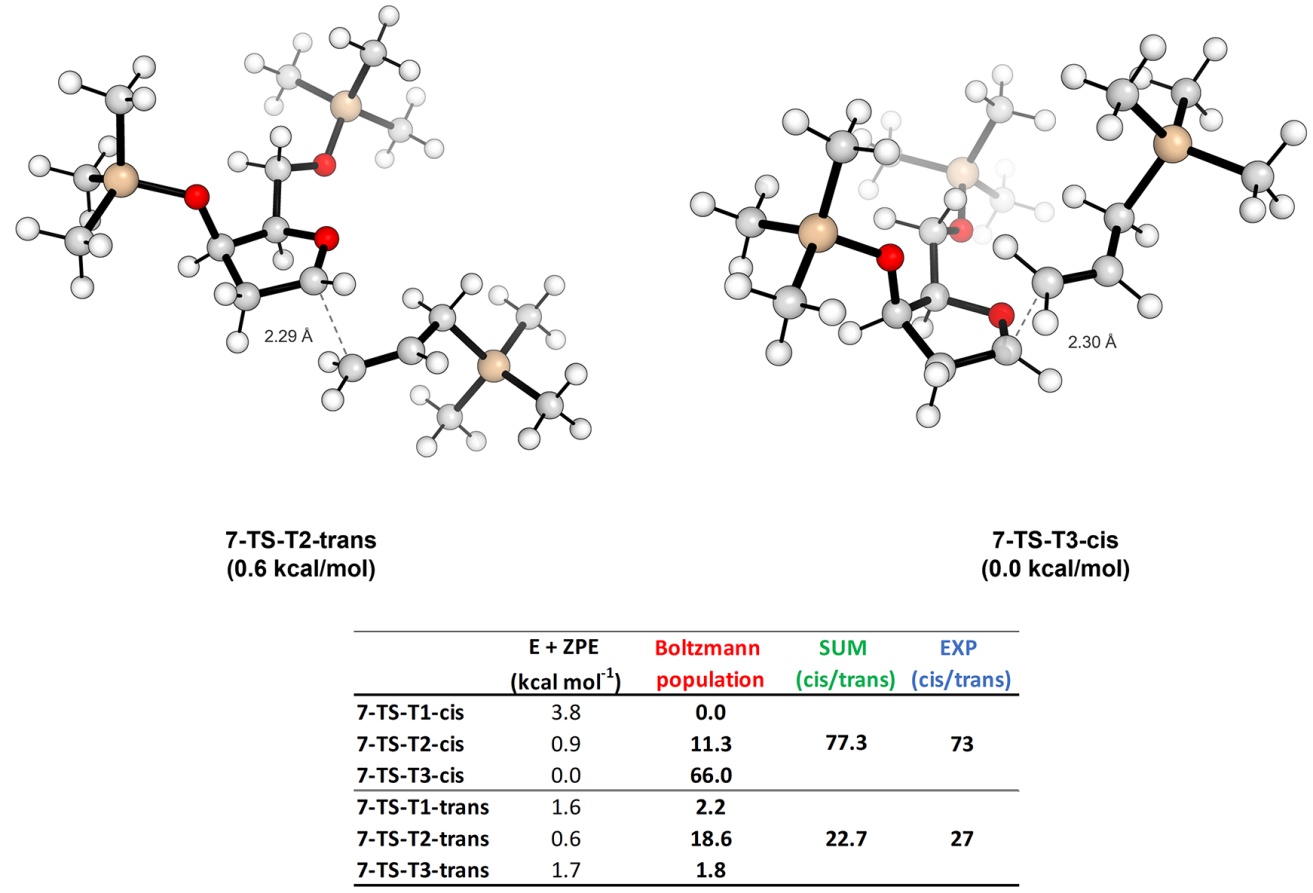
TSs for the trans- and cis-approach of the allyltrimethylsilane on **7** and the table reporting the Boltzmann percentage population evaluated at −30 °C for all possible conformations.

In particular, a mismatch can be predicted in the case of the cis-4,5-disubstituted oxo-carbenium ion **7**. The DFT calculations predicted the all-cis trisubstituted THF product as the preferred diastereoisomer but with a lower selectivity than in the case of **6**. Indeed, the calculated cis/trans ratio (considering all possible orientations on the C-5 silylether) is 77.3:22.7, in good agreement with the available experimental data from our reactions, which show a diastereomeric ratio of 73:27.^5^ The most stable TSs for the cis and trans allylation of oxo-carbenium ion **7** (shown in Figure 4) differ by 0.6 kcal/mol at −30 °C. In both the approaches, the C-4 silylether group is pseudoaxially oriented as in **6**, but the effect it exerts is directed toward the side chain at C-5. Indeed, its vicinal presence forces the C-5 silylether in the pseudoequatorial orientation, outside the heterocyclic ring, favoring the **T3** conformation and thus reducing the contrast in the approach of nucleophile toward the cis-allylation. Vice versa, the trans-approach prefers a **T2** conformation, given the impossibility of orienting the oxygen of the side chain at C-5 above the ring and close to the oxonium center like in oxo-carbenium **5**. The pseudoequatorial orientation remains the most favored also for the trans-approach. Since the orientations of the substituents are strongly different with respect to the previous mono-substituted cases, we should expect a strong variation in terms of puckering parameters. This is particularly evidenced in the trans-approach, where the **T1** conformation assumes the ^*3*^*T*_*4*_ puckering as for **5**, but the other most populated one (namely, **T2**) shows the ^*4*^*T*_*5*_ puckering, like in the previous trans-allylation in **6**. Regarding the cis-approach, the ^*4*^*T*_*3*_ puckering remains again the most favored (see Table S6 in the Supporting Information for more details). It thus seems that the strong role in controlling the puckering derives from the orientation of the substituent in C-4, rather than the one in C-5, in accordance with the 1,3-selectivity (2,4-one with our numbering system) described by Woerpel and co-workers.^19^ This observation can also be understood by considering a stabilizing H-bond interaction between the polarized terminal vinylic proton and the oxygen atom at C-4, which is present only if the approach is on the same side of the substituent at C-4.

As a final observation, the distance between the oxo-carbenium C-2 carbon and the terminal allylic one resulted to be similar to the ones reported for **6** (∼2.3 Å).

**Figure 5:**
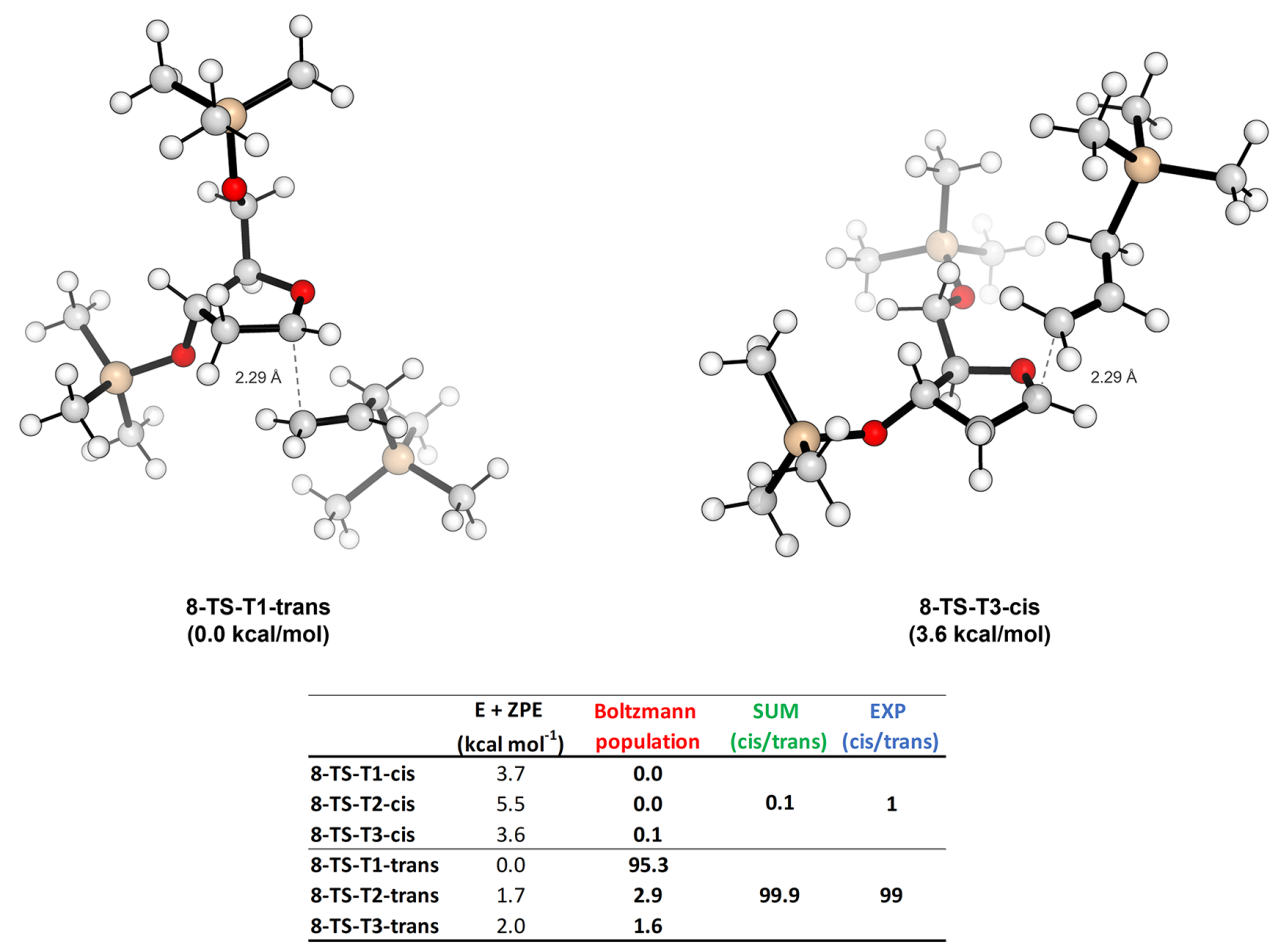
TSs for the trans- and cis-approach of the allyltrimethylsilane on **8** and the table reporting the Boltzmann percentage population evaluated at −30 °C for all possible conformations.

Differently from oxo-carbenium **7**, the opposite orientation of the substituent at C-4 in **8** should reinforce the 1,3-orienting effect (2,4-in our numbering system) in the allylation of the *trans*-4,5-disubstituted oxo-carbenium ion. According to our experimental results and calculations, synergistic cooperation of the C-4 and C-5 trans-substituents allows almost complete diastereoselectivity. The calculated energy difference between the two most populated TSs is 3.6 kcal/mol, corresponding to a 99.9:0.1 ratio at −30 °C between the trans- and cis-products. This result is in good agreement with the experimental value of 99:1 determined for the allylation of **8** as reported in our recent publication.^5^ The calculated most stable TSs for the cis and trans products are shown in Figure 5. Similar to the last case, here also, we can analyze the orientation of the substituents already installed on the oxo-carbenium ring in detail. In the trans-approach, the **T1** conformation remains the most stable one, with both the substituents oriented in pseudoaxial positions. The puckering remains ^*3*^*T*_*4*_, the classically favored one for the trans-approach that we observed also in the above oxo-carbenium **5** (see Table S7 in the Supporting Information for more details). Regarding the cis-approach, ^*4*^*T*_*3*_ puckering is assumed only in the most preferred TS conformer (i.e., **T3**) by showing now a general behavior in bringing the substituents in a bis-pseudo equatorial position. However, the bis-pseudoaxial one remains still possible in the other less populated conformers (i.e., **T1** and **T3**), where ^*5*^*T*_*4*_ puckering is now preferred (see Table S7 in the Supporting Information for more details). Nevertheless, the stabilization provided by the interaction between the oxo-carbenium moiety and the oxygen of the side chain above the ring is not still strong enough to revert the selectivity like in the first case we analyzed with oxo-carbenium **5**. Moreover, also in this case, the stabilizing H-bond interaction between the polarized terminal vinylic proton and the oxygen atom at C-4 appears favorably, which now directs toward the trans-approach. Finally, the distance between the anomeric carbon and the terminal allylic one resulted to be in all the cases similar to the previously reported one (∼2.3 Å).

## Conclusions

In conclusion, DFT computational studies were able to assist in the unveiling of the energetic profile for the rhenium-catalyzed allylation reaction of five-membered ring acetals. Importantly, critical steps of the reaction including the rate-determining step in the ionization toward the formation of oxo-carbenium were revealed. Moreover, all the TSs of the diastereoisomeric allylation step were located by showing the key factors which govern the stereoselectivity of the reaction independently from the anomeric configuration of the starting acetal. The theoretical data supports the experimental results reported by Woerpel and co-workers and is proven also in our recent publication.^3,5,19,20^ In particular, we confirmed the conclusions advanced by Woerpel, where the diastereoselection in C-glycosylation reaction of ribose-derived analogues mainly arises from the relative orientation of the substituent at C-4, given the only marginal, although not negligible, the contribution of the one at C-5.

## Experimental Section

### Computational Methods

For the catalytic cycle, all the structures were optimized using the Gaussian 09 program package,^8^ using the B3LYP functional and a differentiated basis set according to the atom type.^9^ Specifically, 6-311+G(d,p) was used for H, C, O, and F^10,11,18^ and 6-311+g(2df,p) for P, S, and Cl.^11,18^ The effective core potential basis set LanL2DZ was used for Re and Cu atoms.^12^ Calculations performed in presence of Cu species were run using UB3LYP, to fulfill the doublet nature of the spin state for Cu(II) species. All the optimizations were performed in vacuo. Frequency calculations were performed for each species in order to confirm the effective minimum or TS nature of the optimized structures. Moreover, IRC calculations were also used to confirm that all TS structures are linked with that of the reactant, intermediate, or products.^13^ With the optimized geometries, single-point energy calculations in dichloromethane were performed using the PCM^14^ and the same basis set used in the optimization step. All data reported for the catalytic cycle are referred to this level of theory. Moreover, Cremer–Pople conformational analysis was performed in order to identify the puckering parameters which describe the tetrahydrofuran ring as a result of the structural stability.^15^

Regarding the computational investigation of the diastereoselectivity in the alkylation step, we adopted a similar level of theory. We located and optimized the TSs in the reaction step with allyltrimethylsilane by always using the B3LYP functional and a different basis set: 6-311+G(d,p) was used for H, C, and O atoms,^10,11a,18a^ while 6-311+G(2df,p) was used to account for the Si atom.^10,11b−11d,18b^ Tight convergence criteria for the SCF cycles have been adopted (*SCF = tight*). Moreover, the effect of the solvent dichloromethane was directly considered during the optimizations by means of the PCM.^14^ Frequency calculations to extract thermochemical parameters and corrections were performed at the specific reaction temperature reported in the papers, and the theoretical product distributions were obtained by applying Boltzmann population analysis.^5,19,20^ Conformational analysis for the gauche orientations of the substituent around the CH_2_-chain at the C-5 position was manually performed by identifying three possible relative minima as conformers **T1**, **T2**, and **T3**. Finally, we decided to simplify the TBS or TBDPS moieties with a computationally cheaper TMS group.

## Supporting Information Available

Benchmark with different levels of theory; conformational and structural features of the substituted oxo-carbenium species; tables with energies, distances, and puckering parameters for the alkylation of the oxonium species; puckering polar plot; and summary of energies for the optimized structures (PDF)
Cartesian coordinates of the structures, together with the value for the electronic energy and frequencies for the optimized species (XYZ)

## Supplementary Material

jo2c00393_si_001.pdf

jo2c00393_si_002.xyz
