# Supplementary material for: Oxo-Rhenium-Mediated Allylation of Furanoside Derivatives: A Computational Study on the Mechanism and the Stereoselectivity
Source: J Org Chem. 2022 Jul 12;87(15):9497–506. doi: 10.1021/acs.joc.2c00393 (PMC9361356; doi:10.1021/acs.joc.2c00393)
Supplement: Supplementary file 1 — jo2c00393_si_001.pdf [file jo2c00393_si_001.pdf]

Supporting information

Oxo-rhenium mediated allylation of furanoside derivatives: A computational study on the mechanism and the stereoselectivity

Emanuele Casali*, † Alessio Porta, † Lucio Toma † and Giuseppe Zanoni* †

† *Department of Chemistry, University of Pavia, Via Taramelli 12, Pavia 27100 (Italy)*

e-mail corresponding autors: gz@unipv.it emanuele.casali@unipv.it

Benchmarking studies page S2

Table S1 page S2

Figure S1 page S3

Table S2 page S3

Table S3 page S4

Table S4 page S4

Table S5 page S4

Table S6 page S5

Table S7 page S5

Puckering polar plot page S6

References page S6

Table summary of energies for optimized structures (catalytic cycle) page S8

Table summary of energies for optimized structures (stereoselectivity) page S9

Benchmarking studies

The proper level of theory to describe the system was selected as the best compromise between accuracy and computational cost. We decided to use as a benchmark, the rate determining step (RDS) of the catalytic cycle, corresponding to the one regulated by **TS-2**. The performed calculations were carried out with Gaussian09 software,[1] by applying four level of theory within the two functional B3LYP[2] and PBE0.[3] The choice for those two functionals derives from a computational paper appeared in 2012 which deeply analyze the electronic structures of oxo-Re(V) complexes with the functionals above reported.[4] However, since our system is different – in terms of complexity – from the ones there reported, we decided to reinvestigate both of the two functionals and different combinations of basis sets (*vide infra*). Firstly we approached the RDS with a low level of theory (Table S1-A) which consist in the B3LYP functional with a differentiate basis set: 6-31+G(d,p) for H, C, O, P, Cl[5] and LanL2DZ for Re.[6] We then moved towards two higher levels of theory, by remaining again within the B3LYP functional. Specifically, we tested the differentiated triple-ζ basis sets 6-311+G(d,p) on H, C, O[7] and 6-311+G(2df,p) on P and Cl,[5,8] by again maintaining LanL2DZ on Re (Table S1-B). Moreover, we treated also H, C, O, P, Cl with the Ahlrichs Def2TZVP[9] and Re with the SDD Stuttgart-Dresden ECP basis set (Table S1-C).[10] The three reported levels of theory performed equally well in the case under study, by reproducing in a similar way the E+ZPE for the RDS. An additional test was performed under the PBE0 functional. Since the three approaches adopted with B3LYP resulted in similar results, we decided to test the PBE0 functional with the basis set B, consisting in the best compromise between computational cost and accuracy (Table S1-D). The results we obtained are again in accordance with the previous cases, thus resulting in overall standard deviations within 2.5 kcal/mol. Based on these results we selected the B approach as the election theory to generate the optimized electronic structures for all the catalytic cycle.

	**A**	**B**	**C**	**D**	**Average**	**Std. Dev.**
**2**	0.0	0.0	0.0	0.0	0.0	0.0
**TS-2**	24.2	24.6	24.2	29.3	25.6	2.5
**C + 3 Adduct**	16.1	15.6	16.4	17.2	16.3	0.7

**A**	**B3LYP/6-31+g(d,p)&LanL2DZ**
**B**	**B3LYP/6-311+g(d,p)&6-311+g(2df,p)&LanL2DZ**
**C**	**B3LYP/Def2TZVP&SDD**
**D**	**PBE0/6-311+g(d,p)&6-311+g(2df,p)&LanL2DZ**

Table S1: *Benchmarking studies of the RDS with different basis set and DFT functionals. The reported values are gas-phase E+ZPE (kcal/mol) relative to intermediate* ***2****.*

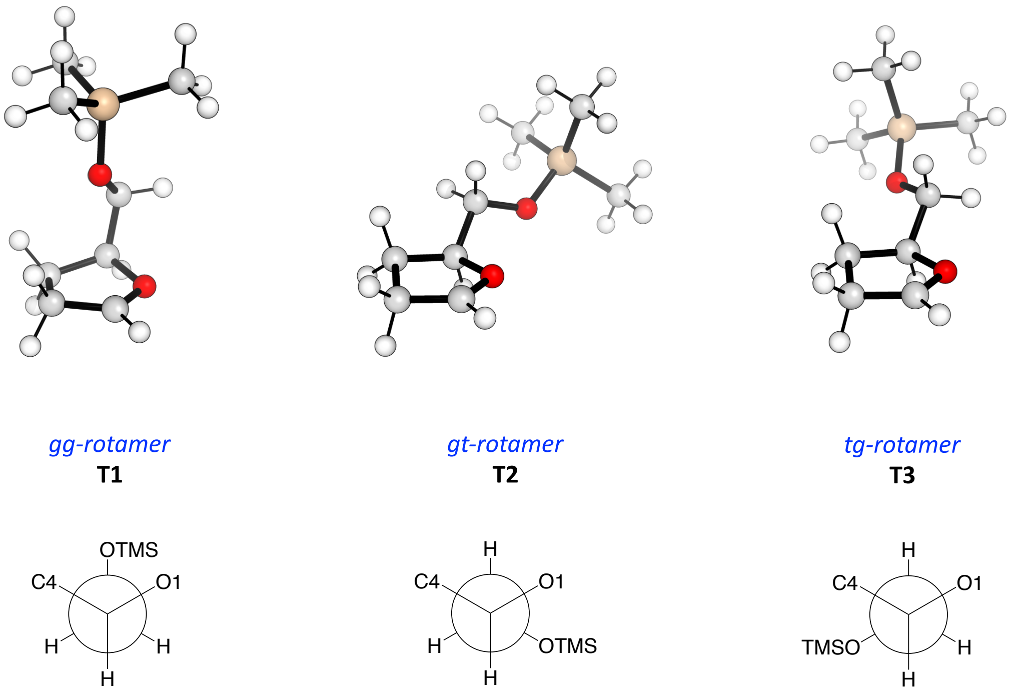

Figure S1: *Examples of rotamers* ***T1, T2*** *and* ***T3*** *around the C-5/CH_2 exocyclic bond of oxo-carbenium* ***5*** *(structures and Newman projections).*

	Puckering Parameters		
	q_2 (Å)	φ_2 (°)	Conformational descriptor
**1**	0.356	69.5	^{3}E
**TS-1**	0.337	78.9	^{3}E
**2**	0.335	71.5	^{3}E
**TS-2**	0.251	298.4	$^{4}T_{5}$ / ^{4}E
**3**	0.176	291.4	^{4}E

Table S2: *Puckering parameters for the heterocyclic species involved in the catalytic cycle.*

	E_{rel} (kcal/mol)	$E+ZPE_{rel}$ (kcal/mol)	G_{rel} (kcal/mol)	$d_{C2\text{-}C(allyl)}$ (Å)	$d_{C\text{-}Si(TMS)}$ (Å)	Puckering Parameters		
						q_2 (Å)	φ_2 (°)	Conformational descriptor
**3-allyl-TMS**	0.0	0.0	0.0	2.79	1.92	0.221	100.1	E_4 / $^3T_4$
**TS-5**	0.7	1.1	1.7	2.29	1.95	0.248	87.8	$^3T_4$
**4**	-10.6	-8.5	-8.3	1.55	2.15	0.357	125.5	$^5T_4$

Table S3: *Energies, distances and puckering parameters for the alkylation of the unsubstituted oxo-carbenium* ***3*** *(calculations performed at -30°C).*

	E_{rel} (kcal/mol)	$E+ZPE_{rel}$ (kcal/mol)	G_{rel} (kcal/mol)	$d_{C2\text{-}C(allyl)}$ (Å)	Puckering Parameters		
					q_2 (Å)	φ_2 (°)	Conformational descriptor
**5-TS-T1-cis**	0.6	0.8	1.6	2.26	0.262	270.9	$^4T_3$
**5-TS-T2-cis**	1.2	1.2	1.1	2.24	0.278	275.1	$^4T_3$
**5-TS-T3-cis**	2.0	2.4	2.6	2.29	0.273	275.2	$^4T_3$
**5-TS-T1-trans**	0.0	0.0	0.0	2.23	0.228	85.0	$^3T_4$
**5-TS-T2-trans**	1.1	1.3	1.4	2.28	0.202	75.7	^3E
**5-TS-T3-trans**	1.9	1.8	1.7	2.31	0.216	82.4	^3E / $^3T_4$

Table S4: *Energies, distances and puckering parameters for the alkylation of the oxo-carbenium* ***5*** *for all the possible conformations (calculations performed at 22°C).*

	E_{rel} (kcal/mol)	$E+ZPE_{rel}$ (kcal/mol)	G_{rel} (kcal/mol)	$d_{C2\text{-}C(allyl)}$ (Å)	Puckering Parameters		
					q_2 (Å)	φ_2 (°)	Conformational descriptor
**6-TS-cis**	0.0	0.0	0.0	2.36	0.258	272.9	$^4T_3$
**6-TS-trans**	1.5	1.9	2.8	2.32	0.257	315.2	E_5 / $^4T_5$

Table S5: *Energies, distances and puckering parameters for the alkylation of the oxo-carbenium* ***6*** *(calculations performed at 23°C).*

	E_{rel} *(kcal/mol)*	*$E+ZPE_{rel}$* *(kcal/mol)*	*G_{rel}* *(kcal/mol)*	*$d_{C2\text{-}C(allyl)}$* *(Å)*	***Puckering Parameters*** *q_2* (Å)	φ_2 (°)	*Conformational descriptor*
**7-TS-T1-cis**	3.6	3.8	4.7	2.32	0.284	276.5	$^4T_3$
**7-TS-T2-cis**	1.0	0.9	0.9	2.34	0.292	278.7	^4E / $^4T_3$
**7-TS-T3-cis**	0.0	0.0	0.0	2.30	0.282	276.7	$^4T_3$
**7-TS-T1-trans**	1.6	1.6	1.2	2.25	0.264	95.2	$^3T_4$
**7-TS-T2-trans**	0.8	0.6	0.1	2.29	0.299	308.7	$^4T_5$
**7-TS-T3-trans**	2.0	1.7	0.6	2.33	0.317	305.9	$^4T_5$

Table S6: *Energies, distances and puckering parameters for the alkylation of the oxo-carbenium* ***7*** *for all the possible conformations (calculations performed at -30°C).*

	E_{rel} *(kcal/mol)*	*$E+ZPE_{rel}$* *(kcal/mol)*	*G_{rel}* *(kcal/mol)*	*$d_{C2\text{-}C(allyl)}$* *(Å)*	***Puckering Parameters*** *q_2* (Å)	φ_2 (°)	*Conformational descriptor*
**8-TS-T1-cis**	3.2	3.7	5.7	2.28	0.190	121.4	$^5T_4$
**8-TS-T2-cis**	5.2	5.5	7.0	2.27	0.179	131.2	$^5T_4$
**8-TS-T3-cis**	3.3	3.6	5.1	2.29	0.247	273.3	$^4T_3$
**8-TS-T1-trans**	0.0	0.0	0.0	2.29	0.246	91.4	$^3T_4$
**8-TS-T2-trans**	1.6	1.7	2.6	2.34	0.217	82.0	^3E / $^3T_4$
**8-TS-T3-trans**	2.1	2.0	3.3	2.40	0.247	93.0	$^3T_4$

Table S7: *Energies, distances and puckering parameters for the alkylation of the oxo-carbenium* ***8*** *for all the possible conformations (calculations performed at -30°C).*

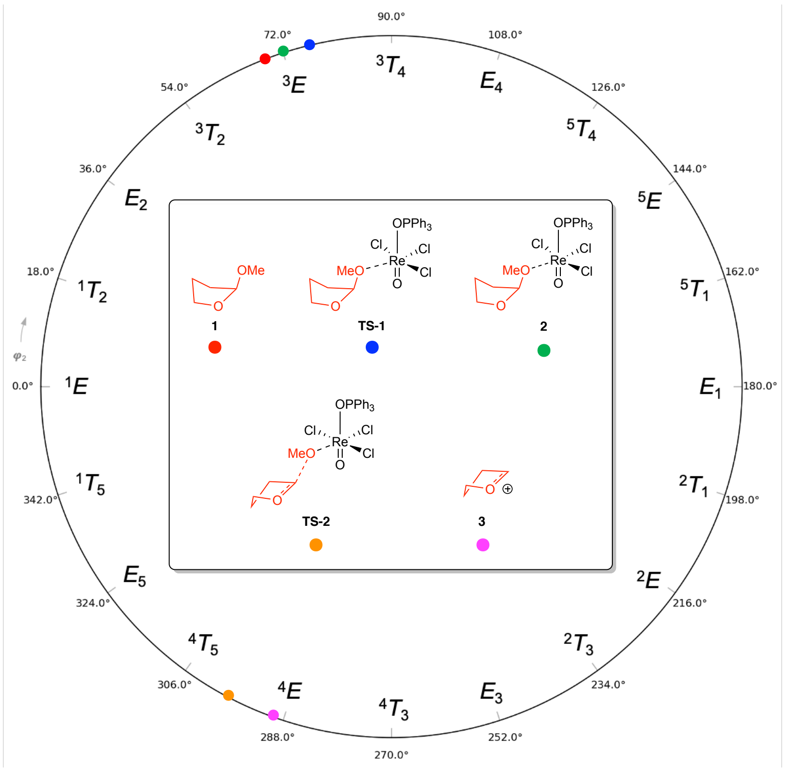

Puckering polar plot: *Puckering parameters in polar coordinates for the evolution of the five-membered ring of **1** during the catalytic cycle.*

References

[1] Frisch, M. J.; Trucks, G. W.; Schlegel, H. B.; Scuseria, G. E.; Robb, M. A.; Cheeseman, J. R.; Scalmani, G.; Barone, V.; Mennucci, B.; Petersson, G. A.; Nakatsuji, H.; Caricato, M.; Li, X.; Hratchian, H. P.; Izmaylov, A. F.; Bloino, J.; Zheng, G.; Sonnenberg, J. L.; Hada, M.; Ehara, M.; Toyota, K.; Fukuda, R.; Hasegawa, J.; Ishida, M.; Nakajima, T.; Honda, Y.; Kitao, O.; Nakai, H.; Vreven, T.; Montgomery, J. A., Jr.; Peralta, J. E.; Ogliaro, F.; Bearpark, M.; Heyd, J. J.; Brothers, E.; Kudin, K. N.; Staroverov, V. N.; Keith, T.; Kobayashi, R.; Normand, J.; Raghavachari, K.; Rendell, A.; Burant, J. C.; Iyengar, S. S.; Tomasi, J.; Cossi, M.; Rega, N.; Millam, J. M.; Klene, M.; Knox, J. E.; Cross, J. B.; Bakken, V.; Adamo, C.; Jaramillo, J.; Gomperts, R.; Stratmann, R. E.; Yazyev, O.; Austin, A. J.; Cammi, R.; Pomelli, C.; Ochterski, J. W.; Martin, R. L.; Morokuma, K.; Zakrzewski, V. G.; Voth, G. A.; Salvador, P.; Dannenberg, J. J.; Dapprich, S.; Daniels, A. D.; Farkas, O.; Foresman, J. B.; Ortiz, J. V.; Cioslowski, J.; Fox, D. J. Gaussian 09, Revision B.01; Gaussian, Inc., Wallingford, CT, **2010**.

[2] a) Becke, A. D. Density-functional thermochemistry. III. The role of exact exchange. *J. Chem. Phys*. **1993**, 98, 5648-5652; b) Lee, C.; Yang, W.; Parr, R. G. Development of the Colle-Salvetti correlation-energy formula into a functional of the electron density. *Phys. Rev. B* **1988**, 37, 785-789.

[3] Adamo, C.; Barone, V., Toward reliable density functional methods without adjustable parameters: The PBE0 model, *J. Chem. Phys*. **1999**, 110, 1658-1669.

[4] Demoin, D. W.; Li, Y.; Jurisson, S. S.; Deakyne, C. A., Method and basis set analysis of oxorhenium(V) complexes for theoretical calculations, *Comput. Theor. Chem.*, **2012**, *997*, 34-41.

[5] a) Clark, T.; Chandrasekhar, J.; Spitznagel, G. W.; Schleyer, P. V. R., Efficient diffuse function-augmented basis sets for anion calculations. III. The 3-21+G basis set for first-row elements, Li-F, *J. Comput. Chem*., **1983**, *4*, 294-301; b) Francl, M. M.; Pietro, W. J.; Hehre, W. J.; Binkley, J. S.; Gordon, M. S.; DeFrees, D. J.; Pople, J. A., Self-consistent molecular orbital methods. XXIII. A polarization-type basis set for second-row elements, *J. Chem. Phys.*, **1982**, *77*, 3654-3665; c) Gordon, M. S.; Binkley, J. S.; Pople, J. A.; Pietro, W. J.; Hehre, W. J., Self-consistent molecular-orbital methods. 22. Small split-valence basis sets for second-row elements, *J. Am. Chem. Soc*., **1982**, *104*, 2797-2803; d) Spitznagel, G. W.; Clark, T.; Schleyer, P. V. R.; Hehre, W. J., An evaluation of the performance of diffuse function-augmented basis sets for second row elements, Na-Cl, *J. Comput. Chem*., **1987**, *8*, 1109-1116; e) Ditchfield, R.; Hehre, W. J.; Pople, J. A., Self-Consistent Molecular-Orbital Methods. IX. An Extended Gaussian-Type Basis for Molecular-Orbital Studies of Organic Molecule, *J. Chem. Phys.*, **1971**, *54*, 724-728; f) Hariharan, P. C.; Pople, J. A., The influence of polarization functions on molecular orbital hydrogenation energies, *Theor. Chim. Acta*, **1973**, *28*, 213-222; g) Hehre, W. J.; Ditchfield, R.; Pople, J. A., Self-Consistent Molecular Orbital Methods. XII. Further Extensions of Gaussian-Type Basis Sets for Use in Molecular Orbital Studies of Organic Molecules, *J. Chem. Phys*., **1972**, *56*, 2257-2261.

[6] Hay, P. J.; Wadt, W. R., Ab initio effective core potentials for molecular calculations. Potentials for K to Au including the outermost core orbitals, *J. Chem. Phys*., **1985**, *82*, 299-310.

[7] Krishnan, R.; Binkley, J. S.; Seeger, R.; Pople, J. A., Self-consistent molecular orbital methods. XX. A basis set for correlated wave functions, *J. Chem. Phys*. **1980**, *72*, 650-654.

[8] McLean, A. D.; Chandler, G. S., Contracted Gaussian basis sets for molecular calculations. I. Second row atoms, Z=11-18, *J. Chem. Phys*. **1980**, *72*, 5639-5648.

[9] a) Weigend, F.; Ahlrichs, R., Balanced basis sets of split valence, triple zeta valence and quadruple zeta valence quality for H to Rn: Design and assessment of accuracy, *Phys. Chem. Chem. Phys.*, **2005**, *7*, 3297-3305; b) Weigend, F., Accurate Coulomb-fitting basis sets for H to Rn, *Phys. Chem. Chem. Phys.*, **2006**, *8*, 1057-1065.

[10] Andrae, D.; Haeussermann, U.; Dolg, M.; Stoll, H.; Preuss, H., Energy-adjusted ab initio pseudopotentials for the 2nd and 3rd row transition-elements, *Theor. Chem. Acc.*, **1990**, *77*, 123-141.

Table summary of energies for optimized structures (catalytic cycle)

In the following table we summed up all the electronic and thermochemical data obtained from the optimization of the structures *in vacuo* and followed by the single point calculation in solvent (DCM) for the catalytic cycle.
Moreover, imaginary frequencies for the transition state structures are reported.
Energies and corrections are given in hartrees, while E+ZPE in solvent (DCM) is in kcal/mol.

	E_{gas} (hartree)	$H_{correction\text{-}gas}$ (hartree)	$ZPE_{correction\text{-}gas}$ (hartree)	$G_{correction\text{-}gas}$ (hartree)	$E{+}ZPE_{solvent}$ (kcal/mol)	Imag. Freq. (cm^{-1})
**A**	-3125.067690	0.396096	0.363648	0.296975	-1960795.984	-
**$Cu(OTf)_2$**	-2119.540246	0.075844	0.057498	0.007670	-1330004.486	-
**A+$Cu(OTf)_2$ Adduct**	-5244.645526	0.474663	0.422356	0.323609	-3290815.593	-
**TS-AB**	-5244.619712	0.473185	0.421223	0.322623	-3290804.352	-47.70
**$Cu(OTf)_2$·DMS**	-2597.645107	0.159578	0.134549	0.075007	-1629974.890	-
**B**	-2646.976030	0.312524	0.286099	0.224956	-1660837.549	-
**1**	-347.085720	0.157270	0.149084	0.117458	-217709.007	-
**TS-1**	-2994.057280	0.470991	0.436220	0.365102	-1878539.703	-49.37
**2**	-2994.061835	0.472481	0.437647	0.367956	-1878541.674	-
**TS-2**	-2994.017356	0.467654	0.432345	0.360479	-1878525.566	-77.21
**C + 3 Adduct**	-2994.035333	0.471227	0.435994	0.363736	-1878526.600	-
**3**	-231.632582	0.110623	0.105028	0.077479	-145335.585	-
**TMS^+**	-409.052141	0.115544	0.108311	0.077524	-256664.441	-
**C + TMS^+ Adduct**	-3171.505703	0.477989	0.439350	0.363963	-1989887.878	-
**TS-3**	-3171.492486	0.477820	0.440858	0.369392	-1989876.983	-97.50
**D**	-3171.526996	0.479178	0.441330	0.367973	-1989900.245	-
**TS-4**	-3171.519822	0.477520	0.439590	0.364946	-1989896.718	-41.46
**TMS-OMe**	-524.550341	0.163884	0.152392	0.117276	-329066.944	-

Table summary of energies for optimized structures (stereoselectivity)

In the following tables we summed up all the electronic and thermochemical data obtained from the optimization of the structures *in DCM* for the setereoselectivity studies.
Energies and corrections are given in hartrees and imaginary frequencies for the transition state structures are reported.

	E in DCM *(hartree)*	*H* correction in DCM *(hartree)*	*ZPE* correction in DCM *(hartree)*	*G* correction in DCM *(hartree)*	*Imag. Freq.* **(cm^{-1})**
**3-allyl-TMS**	-758.407738	0.300717	0.287611	0.253129	-
**TS-5**	-758.406689	0.300559	0.288281	0.254814	-162.96
**4**	-758.424616	0.303236	0.290872	0.256749	-

	E in DCM *(hartree)*	*H* correction in DCM *(hartree)*	*ZPE* correction in DCM *(hartree)*	*G* correction in DCM *(hartree)*	*Imag. Freq.* **(cm^{-1})**
**5-TS-T1-cis**	-1281.761103	0.448402	0.421608	0.365361	-181.35
**5-TS-T2-cis**	-1281.760132	0.448271	0.421323	0.363507	-197.57
**5-TS-T3-cis**	-1281.758820	0.448604	0.421813	0.364695	-164.37
**5-TS-T1-trans**	-1281.762083	0.448255	0.421325	0.363783	-202.63
**5-TS-T3-trans**	-1281.760300	0.448451	0.421543	0.364243	-178.91
**5-TS-T2-trans**	-1281.759093	0.448301	0.421227	0.363484	-158.24

	E in DCM *(hartree)*	*H* correction in DCM *(hartree)*	*ZPE* correction in DCM *(hartree)*	*G* correction in DCM *(hartree)*	*Imag. Freq.* **(cm^{-1})**
**6-TS-cis**	-1242.433294	0.418341	0.392432	0.335566	-121.33
**6-TS-trans**	-1242.430836	0.418670	0.392935	0.337524	-166.03

	E in DCM *(hartree)*	*H* correction in DCM *(hartree)*	*ZPE* correction in DCM *(hartree)*	*G* correction in DCM *(hartree)*	*Imag. Freq.* **(cm^{-1})**
**7-TS-T1-cis**	-1765.779719	0.551996	0.526496	0.477144	-151.14
**7-TS-T2-cis**	-1765.783965	0.551859	0.526096	0.475413	-145.80
**7-TS-T3-cis**	-1765.785507	0.551957	0.526276	0.475503	-164.97
**7-TS-T1-trans**	-1765.782938	0.552059	0.526317	0.474809	-191.17
**7-TS-T3-trans**	-1765.784289	0.551860	0.526032	0.474415	-182.08
**7-TS-T2-trans**	-1765.782291	0.551760	0.525814	0.473212	-156.38

	E in DCM *(hartree)*	*H* correction in DCM *(hartree)*	*ZPE* correction in DCM *(hartree)*	*G* correction in DCM *(hartree)*	*Imag. Freq.* **(cm^{-1})**
**8-TS-T1-cis**	-1765.783388	0.552039	0.526356	0.475811	-186.05
**8-TS-T2-cis**	-1765.780229	0.551867	0.526075	0.474708	-194.42
**8-TS-T3-cis**	-1765.783234	0.551923	0.526149	0.474758	-169.00
**8-TS-T1-trans**	-1765.788517	0.551578	0.525627	0.471916	-166.94
**8-TS-T3-trans**	-1765.785959	0.551679	0.525751	0.473462	-129.95
**8-TS-T2-trans**	-1765.785168	0.551352	0.525406	0.473887	-93.21
